# Diagnostic value of carotid intima-media thickness and clinical risk scores in determining etiology of ischemic stroke

**DOI:** 10.1177/23969873231182492

**Published:** 2023-06-19

**Authors:** Esther MW Sievering, Anika Grosshennig, Martina Kottas, Johanna Ernst, Rieke Ringlstetter, Armin Koch, Karin Weissenborn, Gerrit M Grosse

**Affiliations:** 1 Department of Neurology, Hannover Medical School, Hannover, Germany; 2 Institute of Biostatistics, Hannover Medical School, Hannover, Germany

**Keywords:** Atrial fibrillation, carotid intima-media thickness, diagnosis, embolic stroke of undetermined source, stroke etiology

## Abstract

**Background:**

In the general population, carotid intima-media thickness (CIMT) is associated with atherosclerosis as well as atrial fibrillation (AF). However, the extent to which CIMT might be of diagnostic value in clarifying stroke etiology is currently unclear.

**Methods:**

In this retrospective cohort study, we included 800 consecutive patients with acute ischemic stroke. We compared CIMT-values between stroke etiologies. The association between CIMT and cardioembolic stroke was investigated via logistic regression analysis adjusting for vascular risk factors. Receiver operating characteristic analyses were conducted to investigate the diagnostic value of CIMT in comparison to vascular risk factors and clinical AF risk scores (CHA_2_DS_2_VASc, HAVOC, and AS5F).

**Results:**

CIMT-values were highest in patients with cardioembolic or atherosclerotic stroke origin. CIMT was associated with newly diagnosed AF compared against cryptogenic strokes (crude odds ratio (OR) per 0.1 mm-increase of CIMT: 1.26 (95% confidence interval (CI): 1.13–1.41)). After adjustment for vascular risk factors, the effect of CIMT on AF-diagnosis, however, was weakened (adjusted OR: 1.10 (95% CI: 0.97–1.25)). The diagnostic value of CIMT for detection of AF (AUC: 0.60, 95% CI: 0.54–0.65) was outperformed by AF risk scores. Among the scores investigated, the AS5F-score yielded best accuracy and calibration to predict newly diagnosed AF (AUC: 0.71, 95% CI: 0.65–0.78).

**Conclusions:**

CIMT may help in the diagnosis of stroke etiology. However, compared with vascular risk factors or clinical AF risk scores, CIMT does not provide substantial additional information on the risk of newly detected AF. Thus, stratification of AF risk based on scores, such as the AS5F, is advisable.

## Introduction

In up to 30% of all ischemic strokes, the underlying etiology remains cryptogenic.^1^ This lack of knowledge on the actual mechanism comes along with uncertainty in secondary stroke prevention measures with subsequent risk for stroke recurrence. In 2014, an operationalized definition of cryptogenic strokes with an embolic imaging pattern has been introduced as embolic stroke of undetermined source (ESUS).^2^ Three subsequent randomized controlled trials tested the hypothesis that a pragmatic approach of a general oral anticoagulation (OAC) may be superior over platelet inhibition in ESUS,^3–5^ assuming that a majority of ESUS may be due to an inapparent cardiogenic source like atrial fibrillation (AF). Interestingly, these trials remained neutral, indicating that a proper diagnostic work-up is still needed to indicate the appropriate preventive treatment after ischemic stroke.^6^ Moreover, potential embolic sources in cryptogenic stroke, like atrial cardiopathy, non-stenotic atherosclerotic plaques, and others, frequently overlap,^7–10^ further complicating the choice of the appropriate secondary preventive treatment. Additional diagnostic tools that support determination of the actual mechanism of stroke are therefore warranted.

## Aims and hypothesis

In this study, we hypothesized that the carotid intima-media thickness (CIMT) may be useful to support the diagnostic work-up of stroke etiology. CIMT is a measure of subclinical atherosclerosis and was shown to be associated not only with vascular risk factor burden but also with large vessel atherosclerosis, small vessel disease, and AF in the general population, independently from vascular risk factors.^11–16^ According to previous work by our group and others, CIMT may be of value to identify patients with cryptogenic strokes at high risk for newly detected AF or atrial cardiopathy who therefore should undergo intensified heart rhythm analyses.^17,18^ Furthermore, CIMT is easy to measure in a standardized manner via Duplex ultrasound and is commonly integrated into the diagnostic algorithm following stroke. We additionally aimed to clarify whether CIMT may yield additional information on cardiogenic stroke etiology besides current clinical AF scores, that is, CHA_2_DS_2_VASc,^19^ HAVOC,^20^ and AS5F.^21^

## Methods

### Clinical data

This is a single-center retrospective cohort study. A total of 800 consecutive patients treated at the stroke unit of Hannover Medical School between January 2018 and July 2020 were included. All patients provided written informed consent on the use of their data. Individual approval by the local ethics committee was waived due to the applicable regulations and in accordance with the latter. The inclusion criterion was defined as the diagnosis of an acute ischemic stroke (ICD-10: I63.0-9) with evidence by diffusion-weighted imaging (DWI) in magnetic resonance imaging (MRI). During this period, a total of 1883 stroke patients received treatment at our center. In the emergency setting, 329 patients received computed cranial tomography and 471 primary cranial MRI. Five hundred seventy patients underwent an additional type of cranial imaging with 205 CT and 365 MRI scans.

Infarct patterns based on the MRI-DWI scans were evaluated in the course of clinical routine, as well as by two investigators retrospectively for this study. We performed a holistic clinical evaluation of each patient, which included cardiovascular risk factors, that is, arterial hypertension (systolic blood pressure ⩾140 mmHg), diabetes mellitus (HbA_1 c_ ⩾ 6.5%), adiposity (body mass index (BMI) ⩾30 kg/m²), dyslipoproteinemia (LDL ⩾ 116 mg/dl or triglycerides >150 mg/dl), alcohol (no consumption or consumption 1–2 times per week, consumption 3–5 times per week, daily consumption), and nicotine (active smoker, former smoker or no smoking history) consumption and chronic kidney disease (Glomerular Filtration Rate (GFR) <50 ml/min). We determined these risk factors based on available laboratory parameters and the medical history obtained in patient files. We moreover collected information on coagulation disorders, malignant diseases, previous heart valve surgery, and vasculitis in the medical history.

The Doppler and duplex ultrasound examinations performed during the inpatient stay enabled us to determine CIMT of the common carotid artery and to evaluate potentially symptomatic and asymptomatic stenosis of the intracranial and extracranial brain-supplying vessels. CIMT was measured in both common carotid arteries using a semi-automatic system in accordance to the Mannheim Carotid Intima-Media Thickness and Plaque Consensus^22^ and averaged to a mean value for analyses. All measurements were done at one scanner (GE Logiq) and in a standardized way as part of routine clinical care. All digitally stored image files were cross-checked as part of the data collection process.

Based on DWI-MRI images, two examiners independently classified the infarct pattern as embolic or lacunar. Embolic strokes were defined as cortical DWI-hyperintensities, or sub-cortical DWI-hyperintensities with a diameter of at least 2 cm, in accordance with the criteria applied in the RE-SPECT ESUS trial.^3^ The burden of small vessel disease was evaluated using the Fazekas score. CT-angiography or MR-angiography were considered to evaluate presence of intra- and extracranial stenoses and vessel occlusions. In the event of a lack of agreement, regular meetings were held to achieve a consensus.

Long-term ECG and echocardiography studies were used to determine the diagnosis of AF, patent foramen ovale (PFO), and left ventricular function. AF was categorized depending on whether it was previously known or newly diagnosed during hospital stay.

Based on the collected data, two investigators discussed the most likely stroke etiology at hospital discharge according to the Trial of Org 10,172 in Acute Stroke Treatment (TOAST) criteria, that is, large artery atherosclerosis (LAA), small vessel disease (SVD), cardioembolic stroke (CES), other, or cryptogenic stroke. In the subgroup of cryptogenic strokes, ESUS was defined according to the RESPECT-ESUS criteria as follows: Presence of an infarct area in the brain with a non-lacunar distribution pattern, absence of >50% stenosis of an intra- or extracranial vessel supplying the infarct area and absence of a major cardiac source of embolism (e.g. AF). For newly diagnosed AF, we sticked to the “Guidelines for the diagnosis and management of atrial fibrillation” which suggest a 30 second episode of AF to establish the diagnosis.^23^

The CHA_2_DS_2_VASc, HAVOC, and AS5F scores were calculated for patients with cryptogenic or cardioembolic stroke based on the collected data on risk factors. All collected data were recorded in a standardized way using an electronic case report form (eCRF) via OpenClinica (Waltham, MA 02451 USA, Version 3.3).

### Statistical analysis

This was an exploratory study. Categorical data were compared descriptively according to prevalences using absolute and relative frequencies and continuous data using median and interquartile ranges. CIMT values were compared between stroke etiologies using the Kruskall-Wallis test. Univariable and multivariable binary logistic regression models were conducted to examine the influence of CIMT on stroke etiology (CES vs cryptogenic stroke). All multivariable regression models on stroke etiology were adjusted for age (in years), sex, arterial hypertension, diabetes, dyslipoproteinemia, active nicotine consumption, daily alcohol consumption, kidney failure, and obesity (BMI ⩾ 30 kg/m²), since these are presumed confounders of the association between CIMT and stroke etiology. Crude and adjusted odds ratios (cOR / aOR) with respective 95% confidence intervals (CI) were calculated from the logistic regression models. To investigate the diagnostic value of CIMT in comparison to the aforementioned clinical variables as well as clinical AF risk scores, that is, CHA_2_DS_2_VASc, HAVOC, and AS5F receiver operating characteristic (ROC) statistics with corresponding area under curve (AUC) were computed. Box plots, forest plots, and ROC curves were used for graphical depiction. All statistical analyses have been done using SAS Enterprise Guide 7.1 for Windows.

## Results

### Clinical characteristics

A total of 800 patients were included in the analysis. Clinical characteristics of the study cohort are given in Table 1. Of note, as expected, there were substantial differences in the prevalence of vascular risk factor burden depending on stroke etiology. The prevalence of dyslipoproteinemia was highest in LAA while patients with CES revealed arterial hypertension and diabetes mellitus most frequently. The median BMI was highest in patients with CES, these patients also had the highest NIHSS scores on hospital admission. Differences were also observed in home care before hospital admission across stroke etiologies. The majority of patients were 70 years or older, except for strokes of other causes (median age of 50 years). For the diagnosis of cardiac arrhythmias and especially AF, Holter ECG was performed most frequently in the cryptogenic stroke group.

**Table 1.** Clinical and demographic characteristics.

	SVD (*n* = 145)	LAA (*n* = 87)	CES (*n* = 213)	CRY (*n* = 318)	Other (*n* = 23)	Concurrent (*n* = 14)
Age (years)	75 (64.0–81.0)	70 (60.0–79.0)	80 (74.0–85.0)	72 (60.0–80.0)	50 (46.0–62.0)	80 (77.0–82.0)
Sex (female) (%)	61 (42)	29 (33)	111 (52)	138 (43)	7 (30)	5 (35)
BMI (kg/m^2^)	25.3 (23.3–28.6)	25.7 (23.0–28.3)	26.5 (23.4–29.4)	25.6 (23.2–28.0)	26.2 (24.4–29.4)	25.3 (24.2–29.5)
NIHSS at admission	2 [1.0–4.0]	3 [2.0–5.0]	4 [2.0–8.0]	3 [1.0–5.0]	3 [2.0–13.0]	1.5 [1.0–2.0]
CHA_2_DS_2_VASc	5 [4.0–6.0]	4 [4.0–6.0]	6 [5.0–6.0]	5 [3.0–6.0]	2 [2.0–4.0]	6 [5.0–6.0]
ESRS	2 [2.0–3.0]	2 [1.0–3.0]	2 [2.0–3.0]	2 [1.0–3.0]	1 [0.0–1.0]	3 [2.0–3.0]
Holter ECG (%)	115 (79)	72 (82)	59 (27)	271 (85)	15 (65)	5 (35)
Arterial hypertension (%)	117 (80)	60 (68)	184 (86)	221 (69)	9 (39)	12 (85)
Coronary artery disease (%)	13 (8)	5 (5)	37 (17)	29 (9)	0 (0)	4 (28)
Peripheral artery disease (%)	1 (0.6)	5 (5)	10 (4)	14 (4)	1 (4)	1 (7)
Deep vein thrombosis (%)	4 (2)	1 (1)	10 (4)	12 (3)	2 (8)	0 (0)
Pulmonary artery embolism (%)	6 (4)	2 (2)	6 (2)	12 (3)	0 (0)	0 (0)
Dyslipopro-teinemia (%)	96 (66)	69 (79)	142 (66)	221 (69)	14 (60)	10 (71)
Diabetes mellitus (%)	66 (45)	40 (45)	127 (59)	158 (49)	5 (21)	6 (42)
Previously known atrial fibrillation (%)	0 (0)	1 (1)	130 (61)	0 (0)	0 (0)	5 (35)
Myocardial infarction (%)	10 (6)	4 (4)	12 (5)	21 (6)	0 (0)	1 (7)
Heart failure (%)	4 (2)	1 (1)	30 (14)	23 (7)	1 (4)	1 (7)
History of stroke (%)	35 (24)	20 (22)	50 (23)	71 (22)	1 (4)	4 (28)
Kidney failure (%)	24 (16)	12 (13)	57 (26)	53 (16)	0 (0)	5 (35)
History of malignancy (%)	12 (8)	8 (9)	37 (17)	53 (16)	0 (0)	5 (35)
Independence in daily living (%)	108 (74)	72 (82)	148 (69)	260 (81)	20 (86)	13 (92)
Care at home (%)	28 (19)	9 (10)	36 (16)	41 (12)	2 (8)	0 (0)
Nursing home (%)	9 (6)	5 (5)	26 (12)	16 (5)	0 (0)	1 (7)
Nicotine consumption (active smoker) (%)	45 (31)	31 (35)	19 (8)	69 (21)	6 (26)	2 (14)
Nicotine consumption (former smoker) (%)	6 (4)	2 (2)	3 (1)	15 (4)	1 (4)	1 (7)
Alcohol consumption (daily) (%)	10 (6)	6 (6)	14 (6)	22 (6)	1 (4)	2 (14)

SVD: small-vessel-disease; LAA: large artery atherosclerosis; CES: cardioembolic stroke; CRY: cryptogenic stroke; BMI: body mass index; NIHSS: National Institutes of Health Stroke Scale; ESRS: Essen Stroke Risk Score; ECG: electrocardiogram.

Clinical and demographic characteristics of the study cohort. Given are total numbers and percentages (%) for qualitative and medians and interquartile ranges (IQR) for quantitative parameters.

A total of 469 patients (58%) revealed an embolic imaging pattern upon DWI-MRI. An embolic stroke pattern was found in 251 (78%) patients with cryptogenic stroke, 143 (67%) with CES, 51 (58%) with LAA.

### Carotid intima-media thickness and stroke etiology

**Figure 1.**
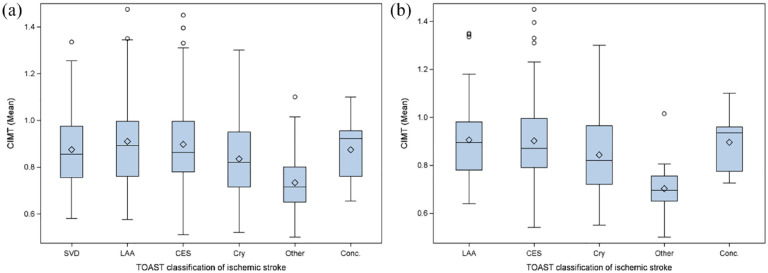
Boxplots depicting the distribution of mean carotid intima-media thickness (CIMT) values in patients with different stroke etiologies: (a) Mean CIMT of the entire cohort with different stroke etiologies according to the Trial of ORG 10,172 in Acute Stroke Treatment (TOAST) criteria, that is, small vessel disease (SVD), large artery atherosclerosis (LAA), cardioembolic stroke (CES), cryptogenic (Cry), other, or stroke with concurrent etiologies (Conc.). *p* < 0.001 according to Kruskall-Wallis test and (b) Comparison of the mean CIMT values between patients who met the criteria for an embolic stroke pattern with different stroke etiologies according to the Trial of ORG 10,172 in Acute Stroke Treatment (TOAST) criteria. *p* < 0.001 according to Kruskall-Wallis test.

As shown in the boxplots in Figure 1(a), mean CIMT values were slightly different between study groups with lowest values in patients with other stroke etiology. The median of the mean CIMT values were lower in patients with cryptogenic stroke versus known stroke etiology. Patients with LAA had the highest mean CIMT values. While in Figure 1(a) the entire patient cohort is depicted, Figure 1(b) presents data of patients who met the criteria for an embolic stroke pattern. Mean CIMT values differed to a larger extent in patients with an embolic stroke pattern as compared to the whole study group.

**Figure 2.**
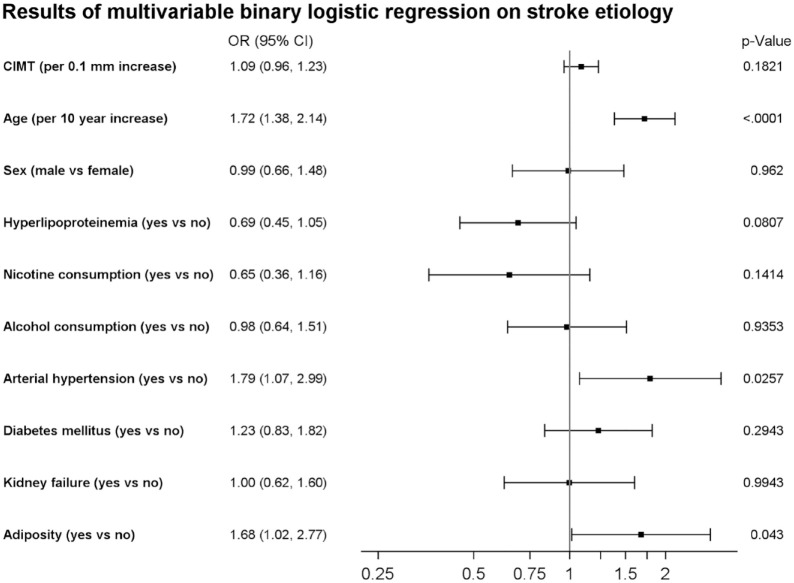
Forest plot indicating adjusted odds ratios (OR) and respective 95% confidence intervals of CIMT and vascular risk factors for stroke etiology (CES vs cryptogenic stroke).

Since it is assumed that paroxysmal AF causes a large proportion of cryptogenic strokes, we analysed whether there are differences in CIMT values between patients with cryptogenic stroke and those with newly diagnosed AF as most likely stroke etiology. In the according crude logistic regression analysis, CIMT was associated with newly diagnosed AF (cOR 1.26 (95% CI: 1.13–1.41)) (see Supplemental Table 1). As depicted in Figure 2, after adjustment for the aforementioned confounding factors the effect of CIMT on AF-diagnosis was considerable smaller (aOR: 1.09 (95% CI: 0.96–1.23)). Instead, age, and arterial hypertension turned out to be the most important risk factors for diagnosis of AF.

Based on the ROC analysis we calculated the Youden’s index which was highest at the CIMT-cutoff of 0.745 mm. Thus, we calculated sensitivity and specificity for AF detection at a measurable cutoff of 0.7 mm which were 93% and 21%, respectively. Therefore, patients with a CIMT below 0.7 mm had a probability not to be diagnosed with AF of 93%.

### Clinical risk scores for AF

**Figure 3.**
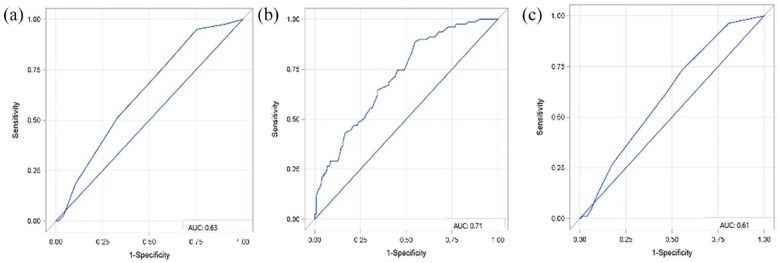
ROC-analyses for comparing the diagnostic value of clinical AF risk scores (CHA_2_DS_2_VASc (a), AS5F (b), HAVOC score (c) in terms of predicting newly diagnosed AF in patients with embolic stroke imaging pattern.

Comparing the value of the CHA_2_DS_2_VASc, the AS5 F and the HAVOC score with each other in terms of predicting newly diagnosed AF in patients with embolic stroke, the AS5F performed best (AUC: 0.71, 95% CI: 0.65–0.78) (Figure 3(b)), in comparison to HAVOC (AUC: 0.61, 95% CI: 0.55–0.67) (Figure 3(c)), and CHA_2_DS_2_VASc (AUC: 0.63, 95% CI: 0.57–0.70) (Figure 3(a)). Calibration of the predictive models was best for the AS5F score (Supplemental Figure 1).

### Carotid intima-media thickness in comparison to risk scores

We in addition compared the diagnostic value for newly diagnosed AF versus cryptogenic stroke by CIMT values with the clinical risk scores HAVOC, CHA_2_DS_2_VASc, and AS5F. For this purpose, we considered the diagnostic value of the individual clinical risk scores, as well as in combination with CIMT values.

The combination of CIMT and HAVOC score did not provide any relevant incremental diagnostic value compared to the HAVOC score alone (ΔAUC: −0.013, 95% CI: −0.014–0.012). Adding CIMT to the CHA_2_DS_2_VASc score in accordance did not reveal any relevant incremental diagnostic value compared to the CHA_2_DS_2_VASc alone (ΔAUC: 0.003, 95% CI: 0.001–0.004). We also did not find any relevant incremental diagnostic value for AS5F in combination with CIMT compared to AS5F alone (ΔAUC: 0.001, 95% CI: −0.002–0.004).

### Small vessel disease burden and CIMT

**Figure 4.**
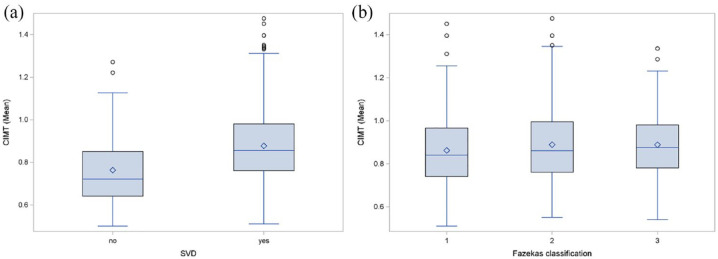
Comparison of the mean carotid intima-media thickness (CIMT) according to prevalence of small-vessel-disease (SVD) (a) and burden of white matter hyperintensities (WMH), as evaluated using the Fazekas classification (b).

Patients with evidence of SVD on MRI (*n* = 709) had a higher median CIMT value (median = 0.86; Q1: 0.76, Q3: 0.98) compared to patients without SVD (median = 0.72 Q1: 0.64. Q3: 0.85) (Figure 4(a)). We compared CIMT values between patients with SVD in regard to the Fazekas classification (Figure 4(b)). Patients with Fazekas grade I revealed a median CIMT value of 0.84 (Q1: 0.74, Q3: 0.97). With increasing SVD the median CIMT values for Fazekas II and III increased (Fazekas II median = 0.86 Q1: 0.76, Q3: 1.00), (Fazekas III; median = 0.88 Q1: 0.78, Q3: 0.98).

The crude logistic regression analysis revealed a positive association between increased CIMT values with prevalence of SVD on MRI (OR per 0.1 mm increase of CIMT: 1.69, 95% CI: 1.41–2.02). However, according to the multivariable analysis adjusting for age and vascular risk factors, this association was largely confounded by age (aOR per 0.1 mm increase of CIMT: 1.11, 95% CI: 0.91–1.35; aOR for age per 10 year-increase: 3.38, 95% CI: 2.55–4.47).

## Discussion

The main results of this study are: (1) CIMT values are higher in patients with a cardiogenic source of stroke as compared to ESUS patients. (2) The diagnostic value of CIMT for AF, however, is outperformed by vascular risk factors and clinical AF risk scores. (3) Among the AF risk scores investigated, the AS5F score yielded best accuracy and calibration to predict newly diagnosed AF in our sample.

A broad body of evidence is available that CIMT, as a measure of subclinical atherosclerosis, is an independent risk factor for AF in the general population.^11,24^ At a pathophysiological level, vascular and left atrial disease are intertwined, since higher vascular stiffness may induce a higher cardiac afterload with subsequent remodeling of the myocardium.^25^ Vice versa, there is also evidence that AF may induce endothelial dysfunction which is regarded as a crucial step in the development of atherosclerotic disease.^26^

Current evidence on ESUS suggests that it is multifactorial disease that may not only be dependent on hidden cardio-embolic sources but also arterio-arterial embolisms and other potential embolic sources.^6,8,10^ To stratify patients according to risks for AF is crucial in this context since OAC in AF has high efficacy in secondary prevention.^27^ On the other hand, intensified rhythm monitoring is time and cost intensive and thus not applicable to the whole spectrum of stroke patients for practical reasons.^28^ Furthermore, biomarkers of atrial cardiopathy may be useful in the future to directly indicate anticoagulant therapy,^29^ as suggested by sub analyses of the ESUS trials.^27^

We were thus interested whether CIMT may be useful to support assignment of stroke etiologies in a well-characterized cohort of patients who suffered imaging-proven acute ischemic stroke. In a previous prospective cohort study, we could show that CIMT and symmetric dimethylarginine (SDMA), which is a biomarker of endothelial dysfunction, are related to AF diagnosed at hospital discharge,^17^ in the long-term course as well as with measures of atrial cardiopathy.^18^ We now aimed to investigate if the association between CIMT and AF is transferrable to stroke etiologies of atherosclerotic origin and to elucidate whether CIMT may yield additional diagnostic information as compared to vascular risk factors and distinct current risk scores that have been developed to predict newly diagnosed AF.

In this current study of consecutively treated patients with imaging-proven acute ischemic stroke, we were able to confirm the association between CIMT and AF. However, the diagnostic accuracy was quite low and did not yield additional value as compared to vascular risk factors. Pruissen et al. and Jin et al. reported higher CIMT values in patients with LAA compared to SVD.^15,30^ This is clearly in line with findings from our cohort, as reflected by the univariable, and multivariable logistic regression analysis. In an analysis of the Northern Manhattan study, CIMT was related to the burden of white matter hyperintensities (WMH), independently from vascular risk factors.^31^ In accordance, in our study, CIMT values were higher in patients with signs of WMH. However, the multivariable analysis revealed that this association was largely confounded by age. In addition, we could not confirm a correlation between CIMT and the burden of WMH, as evaluated using the Fazekas scale in the whole cohort, nor in the subgroup of patients who suffered stroke due to SVD.

The comparison of the diagnostic value of current risk scores for AF with CIMT also showed no advantage for the addition of CIMT values. Of note, the AS5F score yielded the best accuracy and calibration for AF detection when applied to our sample. These results therefore suggest that summation of risk factors in current scores are currently a reasonably good guide for identification of cardiac embolic source. Conversely, it can also be concluded that patients with a narrow CIMT, that is, less than 0.7 mm, are likely to have a low probability of AF, which is relevant in light of the fact that CIMT can relatively easily be obtained as a standard assessment.

This study has several limitations. First of all, despite all efforts to obtain a holistic depiction of clinical variables and risk factors in a standardized way, the retrospective nature of this study clearly comes along with shortcomings in regard to data quality in comparison to a prospective study. However, the criteria collected are rather reliably documented in the patient files, which is also reflected in the fact that we hardly had any missing values. We intended a representation of clinical reality and therefore included all consecutive patients with DWI-MRI positive strokes. However, this in turn results in an imbalance in the sample sizes of different etiologies, since MRI is especially performed in cryptogenic strokes. However, the inclusion criterion of imaging-confirmed stroke is an important benefit in that biasing stroke mimics are excluded and the infarct pattern can be assessed, which is of particular importance in this work. The duration of cardiac monitoring differed between patient groups and it was shown that longer times of monitoring lead to higher detection rates across different stroke etiologies.^32^ All patients were treated at our certified Stroke Unit and therefore received continuous cardiac monitoring and additional Holter ECGs, which was the case for the majority of patients with cryptogenic stroke. Therefore, the risk for an according bias is somehow low. Moreover, duration of cardiac monitoring must not be considered as confounder since it is only a factor for detection of AF and not of CIMT. A particular strength of this work is moreover certainly the high granularity of the data with a sufficiently large sample size, which allowed us to adjust for several covariates.

## Conclusions

CIMT differs among stroke etiologies and is substantially broader in patients with newly detected AF as compared to ESUS patients. However, CIMT does not yield substantial additional information on the risk for newly diagnosed AF in ESUS as compared to vascular risk factors or clinical AF risk scores. In this respect, stratification of the AF risk on the basis of current scores, such as the AS5F score, is advisable.

## Supplemental Material

sj-docx-1-eso-10.1177_23969873231182492 – Supplemental material for Diagnostic value of carotid intima-media thickness and clinical risk scores in determining etiology of ischemic stroke Click here for additional data file. Supplemental material, sj-docx-1-eso-10.1177_23969873231182492 for Diagnostic value of carotid intima-media thickness and clinical risk scores in determining etiology of ischemic stroke by Esther MW Sievering, Anika Grosshennig, Martina Kottas, Johanna Ernst, Rieke Ringlstetter, Armin Koch, Karin Weissenborn and Gerrit M Grosse in European Stroke Journal
