# Supplementary material for: Diagnostic value of carotid intima-media thickness and clinical risk scores in determining etiology of ischemic stroke
Source: Eur Stroke J. 2023 Jun 19;8(3):738–46. doi: 10.1177/23969873231182492 (PMC10472955; doi:10.1177/23969873231182492)
Supplement: sj-docx-1-eso-10.1177_23969873231182492 – Supplemental material for Diagnostic value of carotid intima-media thickness and clinical risk scores in determining etiology of ischemic stroke [file sj-docx-1-eso-10.1177_23969873231182492.docx]

**Online Supplement**

**Supplemental Table 1.** Odds ratios of CIMT and vascular risk factors in diagnosing stroke etiology (CES vs cryptogenic stroke) Reference variable for sex was “female” and for every other category the reference variable was “no”.

|  | Crude Odds Ratio  (95% Confidence Interval) | Adjusted Odds Ratio  (95% Confidence Interval) |
| --- | --- | --- |
| CIMT (per 0.1-mm increase) | 1.26 (1.13-1.41) | 1.09 (0.96-1.23) |
| Age (per 10-year increase) | 1.91 (1.60-2.27) | 1.72 (1.38-2.13) |
| Sex (male vs. female) | 1.42 (1.00-2.01) | 0.99 (0.66-1.48) |
| Hyperlipoproteinemia (yes vs. no) | 0.88 (0.61-1.27) | 0.69 (0.46-1.06) |
| Active nicotine consumption (yes vs. no) | 0.39 (0.25-0.60) | 0.67 (0.42-1.06) |
| Daily alcohol consumption (yes vs. no) | 0.93 (0.84-1.03) | 0.99 (0.89-1.11) |
| Arterial hypertension (yes vs. no) | 2.79 (1.76-4.41) | 1.80 (1.08-3.01) |
| Diabetes mellitus (yes vs. no) | 1.50 (1.05-2.12) | 1.24 (0.84-1.83) |
| Kidney failure (yes vs. no) | 1.83 (1.20-2.79) | 0.99 (0.62-1.59) |
| Adiposity (yes vs. no) | 1.61 (1.05-2.48) | 1.66 (1.01-2.74) |

**Supplemental Figure 1**


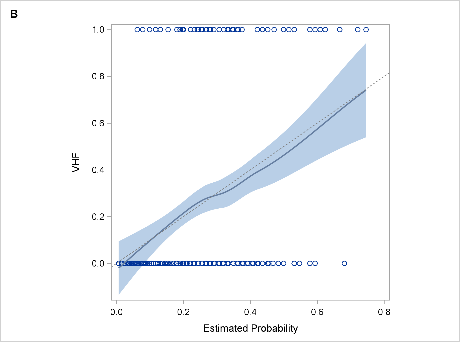

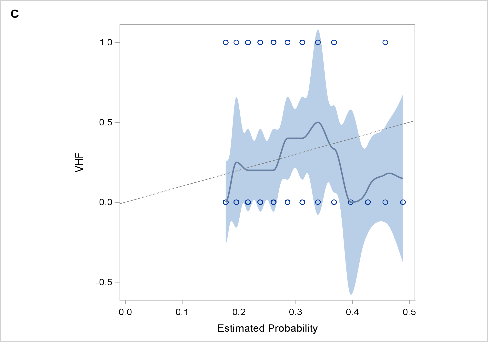

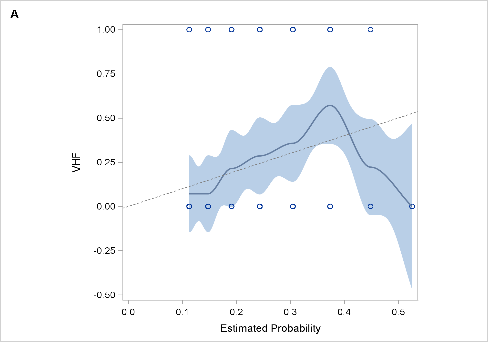


Calibration curves for clinical AF risk scores (**A:** CHA_2_DS_2_VASc, **B:** AS5F, **C:** HAVOC score) in prediction of newly diagnosed AF in patients with embolic stroke imaging pattern.
